# Infusion of HIV-1 Nef-expressing astrocytes into the rat hippocampus induces enteropathy and interstitial pneumonitis and increases blood–brain-barrier permeability

**DOI:** 10.1371/journal.pone.0225760

**Published:** 2019-11-27

**Authors:** Jocelyn Rivera, Raymond A. Isidro, Raisa Y. Loucil-Alicea, Myrella L. Cruz, Caroline B. Appleyard, Angel A. Isidro, Gladys Chompre, Krystal Colon-Rivera, Richard J. Noel

**Affiliations:** 1 HIV-1 Immunopathogenesis Laboratory, The Wistar Institute, Philadelphia, PA, United States of America; 2 Department of Basic Sciences, Ponce Health Sciences University, Ponce Research Institute, Ponce, Puerto Rico, United States of America; 3 Department of Biology, Pontifical Catholic University of Puerto Rico, Ponce, Puerto Rico, United States of America; University of Kentucky Medical Center, UNITED STATES

## Abstract

Even though HIV-1 replication can be suppressed by combination antiretroviral therapy (cART) inflammatory processes still occur, contributing to comorbidities. Comorbidities are attributed to variety of factors, including HIV-1 mediated inflammation. Several HIV-1 proteins mediate central nervous system (CNS) inflammation, including Nef. Nef is an early HIV-1 protein, toxic to neurons and glia and is sufficient to cause learning impairment similar to some deficits observed in HIV-1 associated neurocognitive disorders. To determine whether hippocampal Nef expression by astrocytes contributes to comorbidities, specifically peripheral inflammation, we infused Sprague Dawley rats with GFP- (control) or Nef-transfected astrocytes into the right hippocampus. Brain, lung, and ileum were collected postmortem for the measurement of inflammatory markers. Increased blood-brain-barrier permeability and serum IL-1β levels were detected in the Nef-treated rats. The lungs of Nef-treated rats demonstrated leukocyte infiltration, macrophage upregulation, and enhanced vascular permeability. Ileal tissue showed reactive follicular lymphoid hyperplasia, increased permeability and macrophage infiltration. The intracerebroventricular application of IL-1 receptor antagonist reduced infiltration of immune cells into ileum and lung, indicating the important role of IL-1β in mediating the spread of inflammation from the brain to other tissues. This suggests that localized expression of a single viral protein, HIV-1 Nef, can contribute to a broader inflammatory response by upregulation of IL-1β. Further, these results suggest that Nef contributes to the chronic inflammation seen in HIV patients, even in those whose viremia is controlled by cART.

## Introduction

Combination antiretroviral therapy (cART) has remarkably altered the human immunodeficiency virus type I (HIV-1) epidemic, as cART improves quality of life, can prevent viral transmission, and prolongs the life expectancy of patients living with human immunodeficiency virus type 1 (PLWH)[1]. However, PLWH can still suffer from comorbidities. Neurocognitive impairment as well as cardiovascular, gastrointestinal (GI), and pulmonary diseases pose challenges for managing quality of life of PLWH. Neurocognitive impairment onset can be caused by central nervous system (CNS) inflammation. CNS inflammation can occur early after HIV infection, as the virus is neurotropic and quickly establishes a reservoir in the brain. Macrophages, microglia, and astrocytes are major cell types in brain. These cells are involved in the development of CNS inflammation. Astrocytes are an important, source if viral neurotoxin even when replication is restricted by cART. [2–12]. Astrocytes are susceptible to HIV infection [6, 9, 13, 14] but refractory to viral replication [5, 8]. Hallmarks of brain inflammation can persist even when viral loads are undetectable, in part because of reduced brain penetrance of some antiretroviral drugs, even when peripheral levels achieve therapeutic efficacy [15].

The expression of viral proteins such as Tat, Nef, and GP120 is well documented to induce neuropathogenesis, contributing to the progressive neurological impairment seen very often in PLWH. Of particular interests is Nef, an early HIV protein produced and secreted by infected cells, that it is associated with HIV-associated dementia [16, 17]. Furthermore, microglia or macrophages may transfer Nef to other cells, including those that have not been infected by HIV-1 [18–20]. While cART controls viral replication, it does not prevent the expression of HIV proteins in infected cells [21]. Nef has been shown to downregulate CD4 and MHC I expression, which is thought to contribute to immune evasion by HIV-1 [22–25]. Nef has been shown to be released in exosomes when produced by astrocytes [26] causing neurotoxicity and upregulation of CCL-5 in astrocytes [27, 28].

Brain damage can trigger the pro-inflammatory secretion of cytokines such as, IL-6, CCL-2, and IL-8, that can be released by astrocytes [29].Furthermore, IL-1β and other proinflammatory cytokines released by astrocytes or macrophages/microglia have been identified in the cerebrospinal fluid of HIV patients, suggesting cytokines play an role in HIV-induced CNS pathologies [30, 31]. IL-1β has been implicated in other chronic inflammatory diseases, such as multiple sclerosis and rheumatoid arthritis, and may contribute to the spread of inflammation between the brain and peripheral tissue [32]. For example, in mice with multiple sclerosis and rheumatoid arthritis, high serum IL-1β levels correlate with the elevated CNS expression of IL-1β, IL-8, and TNF-alpha [33]. In plasma, IL-1β expression may contribute to the differentiation of monocytes into macrophages and the acquisition of phagocytic and antigen-presenting properties by macrophages, possibly promoting inflammation in different organs [34].

The development of severe systemic inflammation was shown to be prevalent among traumatic brain injury (TBI) patients [35]. TBI and HIV patients with neurocognitive symptoms correlate with damage to neurons, astrogliosis, and loss of blood-brain barrier (BBB) integrity [36–39]. TBI patients present intestinal mucosa abnormalities, increased gut permeability, and intestinal inflammation [40–42]. These findings suggest a correlation between brain inflammatory processes and pathologies in peripheral organs after TBI. Since the brain is also considered a reservoir for HIV, this raises the possibility that viral or protein activity in the brain will be reflected similarly to peripheral organ inflammation.

Using a rat model in which primary rat astrocytes were implanted in the rat hippocampus and transfected to express Nef, we previously documented the neurocognitive impairment caused by the HIV-1 Nef protein [43]. Although these animals showed normal weight gain, locomotor behavior, and motor coordination, we subsequently observed that rats undergoing the hippocampal infusion of Nef-expressing astrocytes demonstrated gross alterations of the GI tract.

In the present study, we aimed to characterize the effects on the systemic effect that result from the hippocampal implantation of Nef-expressing astrocytes. In light of brain–lung [44, 45] and gut–lung [46, 47] crosstalk and of the effects of HIV-1 infection on the lungs, even in the post-cART era [48], we also examined the effect of astrocytic expression of HIV-1 Nef in the hippocampus on the lungs. Based on the relevance of inflammatory cytokines in driving peripheral inflammation after traumatic injury to the CNS, we also examined the role of IL-1β in our model. In this study, we showed that hippocampal infusion of Nef-expressing astrocytes, induced BBB disruption along with upregulation of IL-1 β in serum. Morphological alteration was observed in lung and ileum. In lung, we found inflammatory cell infiltration (macrophages, eosinophils, and neutrophil) and permeability disruption. Ileal tissue presented enlarged Peyer’s patches, increased macrophage infiltration and permeability disruption. We further characterized that the mechanism involved in the pathologies was dependent on IL1- β, since we demonstrated that blockage of the IL-1 receptor inhibited the pathologies observed.

## Materials and methods

### Institutional Animal Care and Use Committee (IACUC) approval

All experimental protocols involving live animals were pre-approved by the Ponce Health Sciences University Institutional Animal Care and Use Committee (IACUC) protocol number180. The animals were housed in pairs under constant environmental conditions with 12-h light-dark cycle and unrestricted access to food (standard laboratory rat chow) and throughout the study. Pain and discomfort were minimized using inhalation anesthesia for surgical procedures and post-operative application of Neosporin + benzocaine. Animals were observed daily by staff, including veterinary supervision. The parameters to determine the humane endpoint of the study were determined based on the failure to groom, move or feed. None of the rats were excluded from the study.

### Primary rat astrocyte extraction and culture

The extraction and culture of primary rat astrocytes were performed as previously described [43]. Primary rat astrocytes were obtained from the brains of three-month-old Sprague Dawley rats. The rats were anesthetized with pentobarbital and decapitated for brain removal. The brain was minced and disrupted by placing it in ice-cold Hank’s balanced salt solution with trypsin (Sigma, St. Louis, MO). Dulbecco’s Modified Eagle Medium (DMEM, Sigma) containing 10% fetal bovine serum (Corning, Woodland, CA), 10 mM L-glutamine (Sigma), 5% non-essential amino acids (Sigma), and streptomycin/penicillin (Sigma) was added to inactivate the trypsin. The debris was removed from the cells by sedimentation. The process was repeated, discarding the first three extractions to reduce the presence of blood vessels/red blood cells. The cells were pooled and seeded in 75 cm^2^ flasks with DMEM and incubated at 37°C, 5% CO_2_. Non-attached cells were removed after three days in culture. The culture media and conditions were optimized for astrocytes and did not support neuron or microglia survival. The purity of the culture was determined by microscopic analysis and the quantification of astrocytes after immunostaining for the astrocyte marker glial fibrillary acidic protein (GFAP) and counterstaining the nuclei with 4',6-diamidino-2-phenylindole (DAPI). Western blots on cell culture lysates for the microglial marker Iba-1 indicated that no detectable microglia were present in the astrocyte cultures.

### Transient transfection of primary rat astrocytes

Primary rat astrocytes were transfected with a plasmid encoding green fluorescent protein (GFP)-for the control group, or Nef for the experimental group (p96AM651 NIH AIDS Reference Research and Reagent Program, Cat. 8677, donated by Drs. Yingying Li, Feng Gao, and Beatrice H. Hahn). Transfections were performed with a Pulser Xcell (Bio-Rad, Hercules, CA), using 5 μg endotoxin-free DNA plasmid per 1.6 x 10^6^ cells, and pulsed with 250 V for 35 ms. After transfections, cells were resuspended in sterile artificial cerebrospinal fluid (ACSF) at a final concentration of 100,000 cells per 0.5 μL of ACSF.

### Unilateral hippocampal infusion of astrocytes

Thirty-day-old male Sprague Dawley rats were anesthetized by isoflurane inhalation. The skull was exposed, and a hole was drilled in the right parietal bone of the cranium at the following coordinates (using the bregma as reference): anterior-posterior: -0.28 mm; mid-lateral: -0.17 mm; and dorsoventral: -0.37 mm. Transfected astrocytes (100,000) were infused using an injector (33 gauge) with a polyethylene tube (PE-20 from Small Parts, Inc., Logansport, IN) connected to a syringe and mounted on an infusion pump (Harvard Apparatus). After infusion, the skull hole was sealed with bone wax prior to surgical suturing; a triple antibiotic was applied to the stitches to promote recovery.

### Right hippocampal and left ventricle cannulation

For experimental groups treated with IL1-Ra, 30-day-old male Sprague Dawley rats were anesthetized by isoflurane inhalation. The skull was exposed, a hole drilled, and a cannula (Plastics One #C315G-SPC, Roanoke, VA) inserted in the right parietal bone of the cranium at the following coordinates (using bregma as reference): anterior-posterior: -0.28 mm; mid-lateral: -0.17 mm; and dorsoventral: -0.27 mm at a 0° angle to access the right hippocampus. A second hole was drilled and a cannula inserted in the left parietal bone of the cranium at the following coordinates: anterior-posterior: -0.28 mm; mid-lateral: +0.45 mm; and dorsoventral: -0.25 mm at a 12° angle to access the left ventricle. Four surgical screws were placed in the cranium on which to attach dental cement (Dentsply #675571, 675572, York, PA) and prevent the movement of the cannulas and maintain the proper coordinates. The incisions were closed by surgical suturing, and a triple antibiotic plus pain reliever was applied to the stitches to minimize discomfort and promote recovery.

### Infusion of IL1-Ra or saline in the left ventricle and transfected astrocytes in the right hippocampus

Five days after recovery, 5 μL of IL1-Ra (Sigma, 5 μg/mL) or saline (Baxter, Deerfield, IL) were infused through the left cannula using an injector (33 gauge) with a polyethylene tube (PE-20 from Small Parts, Inc.) connected to a syringe and mounted on an infusion pump (Harvard Apparatus). The IL1-Ra dose was based on the inhibition of IL-1-induced sickness syndrome and adjusted for the weight of the animal [49]. The following day, a second, equal dose of IL1-Ra was infused in the left cannula, and 100,000 transfected astrocytes were infused through the right cannula.

### Animal sacrifice

After two days of recovery, the animals were sacrificed with a pentobarbital overdose (1 mL of 65 mg/mL stock per kg) via intraperitoneal injection. When the rats showed no reflexes, the tissues and membranes were cut to expose the heart. Blood was collected by cardiac puncture in the right ventricle. Transcardial perfusions with saline and formalin were done to preserve the remaining tissues.

### Intestinal tissue thickness assessment

The distal ileum tissue was measured with a digital caliper to assess changes in tissue thickness immediately after the rats were sacrificed and before tissue fixation.

### Hematoxylin and eosin staining

The brain, ileum, and lung tissue samples were all fixed in formalin and embedded in paraffin, as previously described [50]. Standard hematoxylin and eosin (H&E, ThermoFisher, Waltham, MA) staining was performed on formalin-fixed and paraffin-embedded tissue. The pathologist examined stained tissues to assess the microscopic and morphologic changes. Peyer’s patch diameter was assessed using a microscope equipped with a calibrated ocular scale. The presence and diameter of Peyer’s patches were analyzed in ileal tissue from four rats per treatment group, for which a total of 33 (GFP) and 42 (Nef) Peyer’s patches were identified and measured. Eosinophils were quantified manually by assessing ten random villi per rat for the presence and number of this leukocyte.

### Myeloperoxidase activity assay

Reagents were from Sigma Aldrich. Unfixed tissue from the distal ileum was dissected and homogenized in potassium phosphate buffer with hexadecyltrimethylammonium bromide and then centrifuged 10,000 rpm for two min. O-dianisidine dihydrochloride and hydrogen peroxide buffer were added, followed by absorbance reading at 460 nm using a BioTek Synergy HT (Bio Tek, Winooski, VT) multimode plate reader.

### CD68, occludin, claudin-5, and CD68 immunofluorescence

Paraffin-embedded brain, ileum, or lung slices (4 μm) were mounted on positively charged slides. The tissue was deparaffinized in xylol (VWR #89370–090,Radnor,PA) and rehydrated in a descending CDA19 ethanol (Thermo Fisher) series, as previously described [50]. Epitope retrieval varied by antibody: For CD68 staining, the slides were incubated with 0.01 M citrate-EDTA (pH = 6.0) for 40 min at 95 to 99°C and followed by 20 min at room temperature; occludin staining was achieved by incubating the slides for 10 min at 37°C with protease (Sigma P-5147); and for claudin-5 staining, the slides were incubated with EDTA for 45 min at 95°C. The slides were incubated overnight in a humidified chamber at 4°C with the primary antibody, mouse anti-CD68 (1:50; AbD Serotec, MCA-341R, Hercules, CA), rabbit anti-occludin (1:100; Invitrogen, 71–1500, Carlsbad, CA), or mouse anti-claudin-5 (1:100; Invitrogen, 35–2500), followed by incubation with Alexa Fluor 555 Goat Anti-Rabbit (Invitrogen, A-21428) for occludin, Alexa Fluor 488 Goat Anti-Mouse (Invitrogen, A-11001) for claudin-5, and Alexa Fluor 594 Goat Anti-Mouse (Invitrogen, A-11032) for CD68 for 30 minutes (1:100). A control reaction was performed without the primary antibody. Tissues were covered with mounting gel and a cover slide. All images were taken using an Olympus microscope and a Nikon digital camera and NIS Elements software (Nikon, Minato, Japan). CD68^+^ cells were quantified using three randomly selected high-power fields (HPFs, image taken at 40x magnification) per rat. Images were taken by an experimenter blinded to treatment groups. CD68 staining was performed in ileum and lung tissues. Tissue florescence analyses were completed using the cell-counter plug-in of the ImageJ software package (version 1.42, NIH, USA).

### Adjusted fluorescence for villus epithelium (AFVE)

AFVE from the epithelium of 10 intestinal villi per rat was quantified using ImageJ software. Fluorescence was measured for seven rats per group using a method adapted to allow for measurement of villi [51]. Integrated fluorescence signals were measured for the entire villus (V) and the villus core (VC); signal for villus epithelium (VE) was calculated by the formula: VE = V–VC. Adjusted fluorescence for villus epithelium (AFVE) was computed by subtracting the background signal from VE, which was then divided by the area of the villus epithelium, to adjust for villus size.

### Ussing chamber assay

Segments of fresh, live ileum were pinned out flat in oxygenated Krebs solution, mucosal side down, in a seven-inch Petri dish coated with SYLGARD (Dow Corning, Midland, MI). The mucosa and submucosa were separated from the outer muscle layers by sharp dissection [52, 53]. The ileum, with the epithelium free of muscularis propria, was mounted between two halves of an Ussing transport chamber that were bathed in warmed, oxygenated Krebs solution. Following a 15-minute equilibration period, the transepithelial potential difference was monitored using a pair of agar-salt bridges connected to a voltage/current clamp (World Precision Instruments, Sarasota, Fl). Transport of ions in any direction of the tissue will produce a voltage difference. This voltage is cancelled out by injecting current. Therefore, the measure of net ion transport will be measure by this short-circuit current (Isc). A pair of current-passing electrodes allowed the determination of basal Isc. At one-minute intervals, the current required to alter the clamping potential was determined, and the tissue resistance was calculated using Ohm’s law. Tissue viability was assessed by the serosal application of acetylcholine (10^−8^–10^−2^ M) with the continual measurement of Isc, which is the charge flow of ions per unit time when the tissue is short-circuited [54]. This approach provided an estimate of the secretory capacity of the tissue. Changes in Isc were used as a measure of net electrogenic ion transport.

### Evans blue assay

Following two days of recovery, 4% Evans blue (EB) dye (Sigma) was injected (400μL/100g) into the tail veins of 10 rats from each group. After two hours, the rats were euthanized with a pentobarbital overdose (1 mL of 65 mg/mL stock per kg). When reflexes were lost, the rats were exsanguinated by transcardial perfusion with 300 mL of saline. The brains and lungs were collected and immersed in formamide (10 mL/g) at 60°C for 48 hours to remove the dye. The absorbance of the supernatant was read using a BioTek Synergy HT multimode plate reader. The EB concentration was calculated by a standard curve that was made using serial dilutions of EB in formamide [55].

### IL-1β ELISA assay

Serum was isolated from blood samples that were collected via a right ventricular puncture during sacrifice. After the collection, the blood was incubated at room temperature for 30 min to allow coagulation. The blood was centrifuged at 10,000 rpm for 10 min to separate the serum from clotted cells. Serum was collected and stored in tubes at -20°C. To assess the levels of pro-inflammatory cytokines in the blood, IL-1β levels were quantified in serum using ELISA (ThermoFisher, cat. ER2IL1B), following the protocol provided by the manufacturer. Samples were run in duplicate. The absorbance was measured using a BioTek Synergy HT multimode plate reader. The IL-1β concentrations were calculated by the interpolated of obtained values on a standard curve.

### Statistical analysis

All statistical analyses were performed using GraphPad Prism, version 8.0 (GraphPad Software, La Jolla, CA). No data were excluded from the analysis any missing data from the animals were solely due to technical reason. Student’s t-test was performed to determine differences between the Nef-treated group and the GFP-treated group (control). One-way ANOVA (followed by Holm–Sidak’s multiple comparisons test) was used to determine the difference between naïve, Nef + placebo, and Nef + IL1-Ra. P values equal to or less than 0.05 were considered significant.

## Results

### Nef expression in the rat hippocampus increased blood-brain-barrier disruption and serum levels of IL-1β

**Fig 1:**
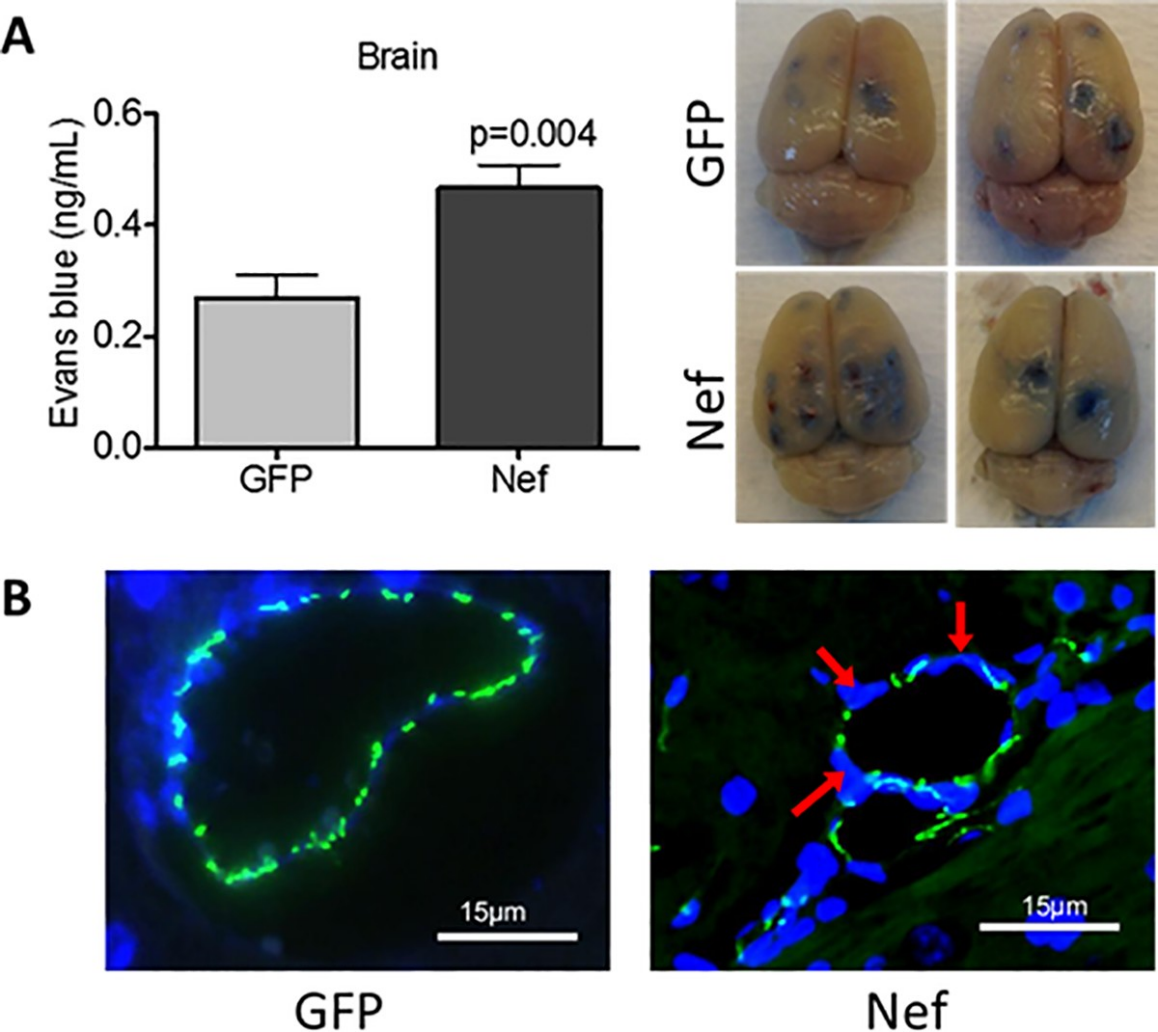
Nef expression in the rat hippocampus disrupts blood-brain-barrier integrity. **(A)** BBB permeability was assessed by injecting EB dye into the tail vein and measuring dye extravasation into the brain tissue. Nef-treated rats showed increased BBB permeability compared to GFP-treated rats. Two whole representative brains for GFP and Nef-treated rats were shown; the infusion was notable in the right hemisphere with increased staining in the Nef group. **(B)** Immunofluorescence of the tight junction protein claudin-5 (green) and nuclei (blue) confirmed BBB disruption (red arrows) in Nef rats. *N* = 9 to 10 rats per group. Scale bars indicate 15 μm. Student’s t-test was performed to determine differences between the Nef-treated group and the GFP-treated group (control). Error bars represent the means ± S.E.M.

**Fig 2:**
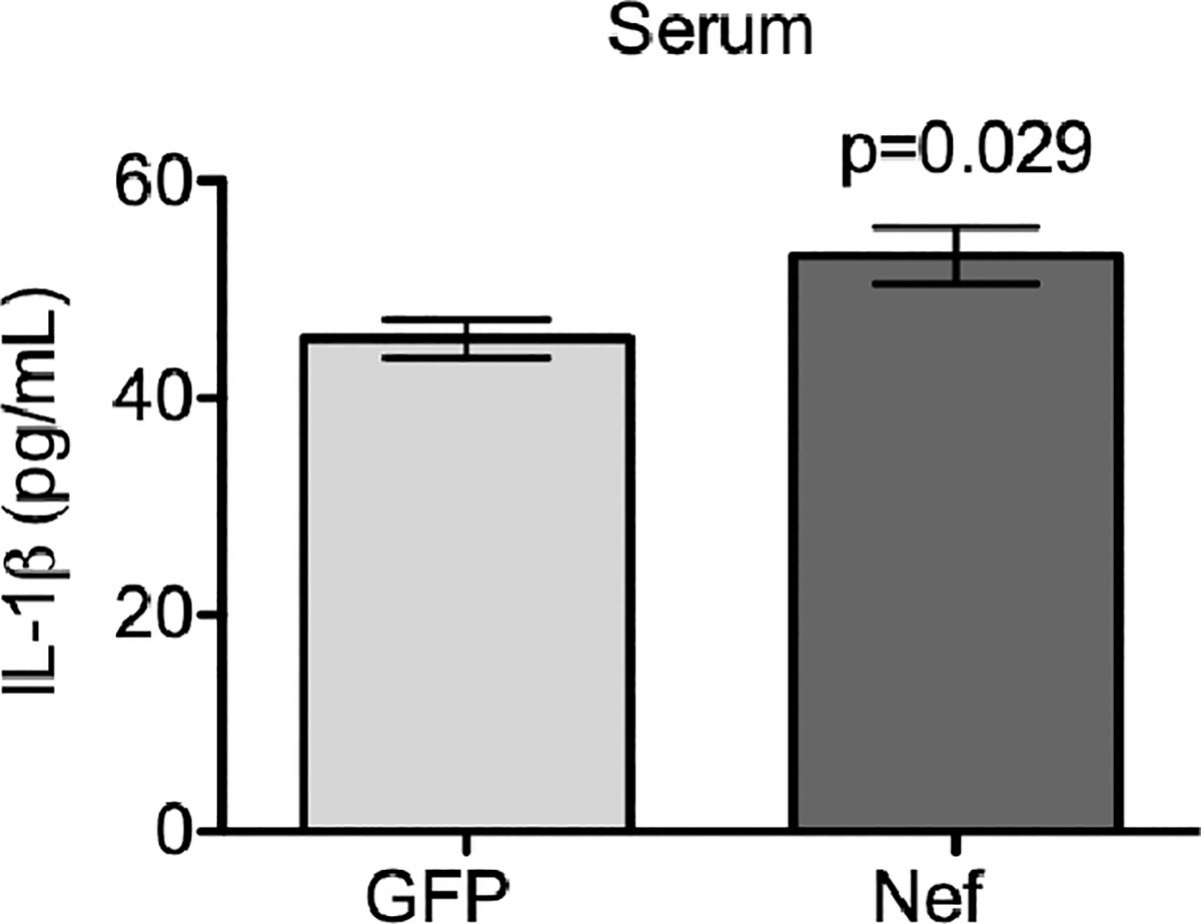
Nef expression in the rat hippocampus increases IL-1β levels in blood. Analysis of IL-1β levels in serum by quantitative ELISA showed that Nef-treated rats had significantly increased levels compared to those treated with GFP. *N* = 9 rats per group. Student’s t-test was performed to determine differences between the Nef-treated group and the GFP-treated group (control). Error bars represent the means ± S.E.M.

Our previous studies demonstrated that the infusion of Nef-expressing astrocytes into the rat hippocampus increased macrophage infiltration at the infusion site compared to what was observed in rats infused with GFP-expressing control astrocytes [43]. Taking into consideration that brain inflammation may induce disruption of the BBB [56, 57], we compared BBB permeability in Nef- and GFP-treated rats by measuring EB extravasation into brain tissue. Nef-treated rats showed significantly increased BBB permeability compared to this in the control group (Fig 1A). BBB integrity disruption was confirmed in Nef animals by immunofluorescence of the tight junction protein claudin-5, which showed a decrease in expression (Fig 1B). Since, Nef has been previously characterized to induce brain inflammation [43] and our current findings of a BBB disruption, we looked at the possibility that the inflammation was systemic. In AIDS patients, serum IL-1β levels correlate with the progression and severity of the disease [58]. To find out whether brain Nef expression increases circulating pro-inflammatory cytokines, we quantified (using ELISA) IL-1β levels in Nef- and GFP-treated rat serum. Compared with their GFP-treated counterparts, Nef-treated rats had significantly elevated serum IL-1β levels (Fig 2). Collectively these results show Nef expression in astrocytes alters endothelial cell tight junctions, barrier function and signs of peripheral inflammation in serum.

### Hippocampal HIV-1 Nef expression causes morphological changes in the ileum

**Fig 3:**
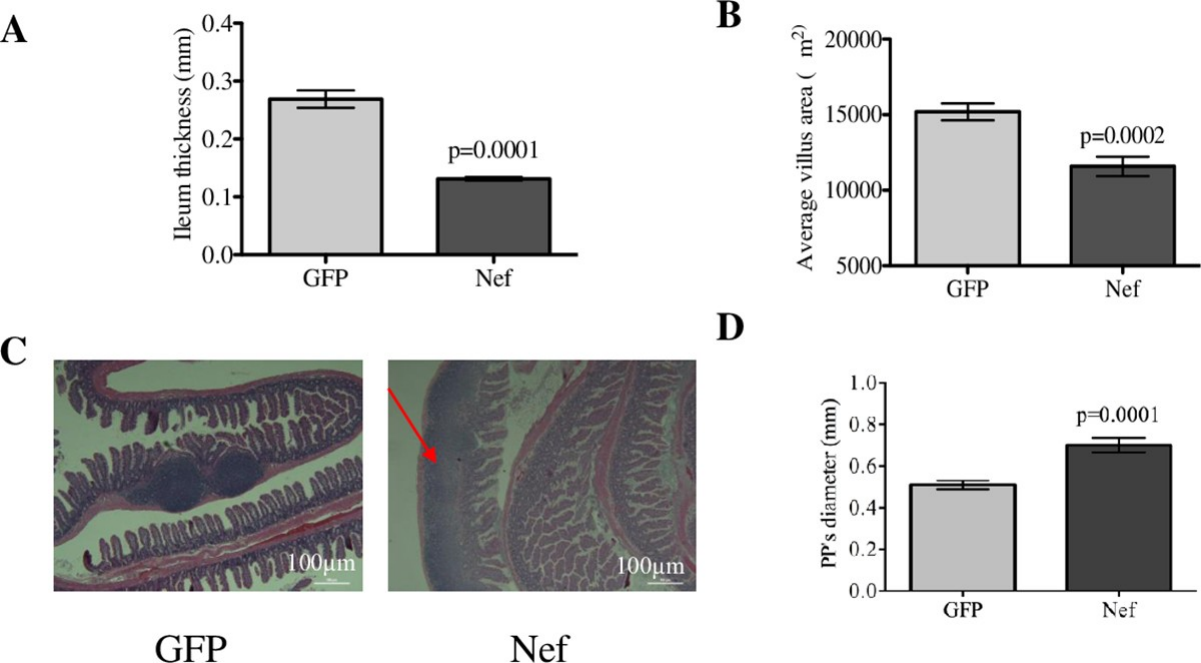
Hippocampal HIV-1 Nef expression decreases ileal tissue thickness and villi area and induces highly reactive follicular hyperplasia. (**A**) Measurements of intestinal thickness with a caliper showed that ileal tissue from Nef-treated rats was significantly thinner than that of the GFP-treated rats. (**B**) Analysis of the villi area showed that the intestinal villi from HIV-1 Nef-treated rats were significantly smaller compared to those of the GFP-treated rats. (**C**) H&E staining of the ileum showed lymphoid hyperplasia (expansion of Peyer’s patches, red arrow) in Nef-treated animals in comparison to normal Peyer’s patches in controls. Scale bars indicate 0.5 mm. (**D**) The microscopic diameters of the Peyer’s patches (PPs) show that PPs from Nef-treated rats are significantly increased compared to those in the GFP-treated rats, confirming that HIV-1 Nef expression in the hippocampus induces highly reactive follicular hyperplasia in the intestines. *N* = 4 to 9 rats per group. Student’s t-test was performed to determine differences between the Nef-treated group and the GFP-treated group (control). Error bars indicate means ± S.E.M.

As previously mentioned, upon sacrificing the rats comprising the study population in our prior study [43], we observed an altered appearance in the intestines of those receiving a hippocampal infusion of Nef expressing astrocytes. To better characterize these changes, we examined the gross and microscopic morphology of the distal ileum in rats with hippocampal HIV-1 Nef or GFP expression. In the Nef group, ileal tissue was ~50% thinner (Fig 3A) and had a friable consistency with a tenacious and increased mucus layer in the bowel mucosa and lumen. H&E staining of paraffin-embedded ileal tissue showed structural villi and muscle layer changes between the Nef- and GFP-treated animals. Microscopic measurements showed that the ileal villi area from the Nef rats was significantly reduced compared to that of the GFP rats (Fig 3B). Ileal tissue in Nef-treated present massive reactive follicular lymphoid hyperplasia found mainly in the mucosal layer and extending to the surface epithelium was observed in Nef animals (Fig 3C). Furthermore, we observed a significant increase in diameters of Peyer’s patches in the Nef-treated when compare to the GFP-treated animals (Fig 3D).

### Inflammatory parameters are increased in the ileum of rats with hippocampal HIV-1 Nef expression

**Fig 4:**
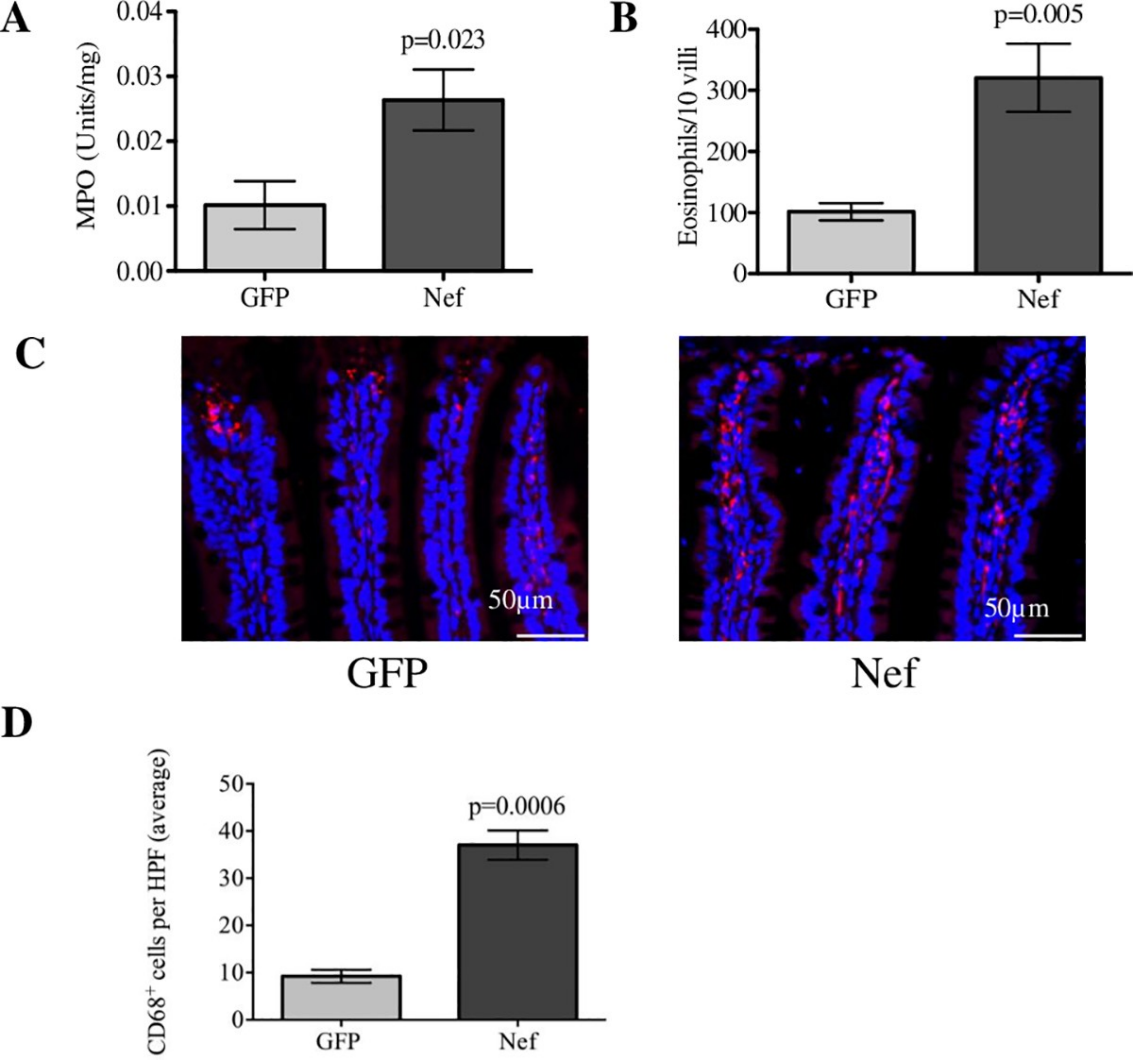
Hippocampal HIV-1 Nef expression induces intestinal inflammation. (**A**) An MPO activity assay was employed to assess myeloid infiltration into the ileal area. Nef-treated rats showed increased MPO levels in the ileum compared to the GFP-treated rats. (**B**) Eosinophils counted from H&E-stained ileal tissue (10 villi per rat) showed that infiltration was significantly increased in the HIV-1 Nef-treated rats. (**C**) Immunofluorescence for the macrophage marker CD68 (red) was assessed to determine the levels of macrophage infiltration between the groups while nuclei of the cell are represented (in blue). (**D**) Macrophages were quantified in three 40x HPFs per rat. *N* = 3 to 7 rats per group. Student’s t-test was performed to determine statistical difference between the Nef-treated group and the GFP-treated group (control). Scale bars indicate 50 μm. Error bars indicate means ± S.E.M.

We next examined several GI inflammatory parameters in rats undergoing infusion of Nef- or GFP-expressing astrocytes into the hippocampus. To determine if Nef expression in a rat brain induced inflammation in the ileum, we measured activity of ileal tissue myeloperoxidase (MPO) as an indirect measure of neutrophil and myeloid cell infiltration. Ileal tissue from Nef rats had 2.5-fold greater expression of MPO than did our control group (Fig 4A; p = 0.023). We also quantified infiltration of eosinophil into the lamina propria of H&E-stained ileal tissue and found that Nef-treated rats had greater than threefold (p = 0.005) increased eosinophil infiltration when compared to the GFP-treated rats (Fig 4B). Macrophage infiltration in the ileal lamina propria was quantified in Nef- and GFP-treated rats using immunofluorescent staining for the macrophage marker CD68 and were found to be significantly increased (fourfold) in Nef-treated rats compared to their GFP-treated counterparts (Fig 4C and 4D).

### Hippocampal HIV-1 Nef expression alters intestinal barrier function in the ileum

**Fig 5:**
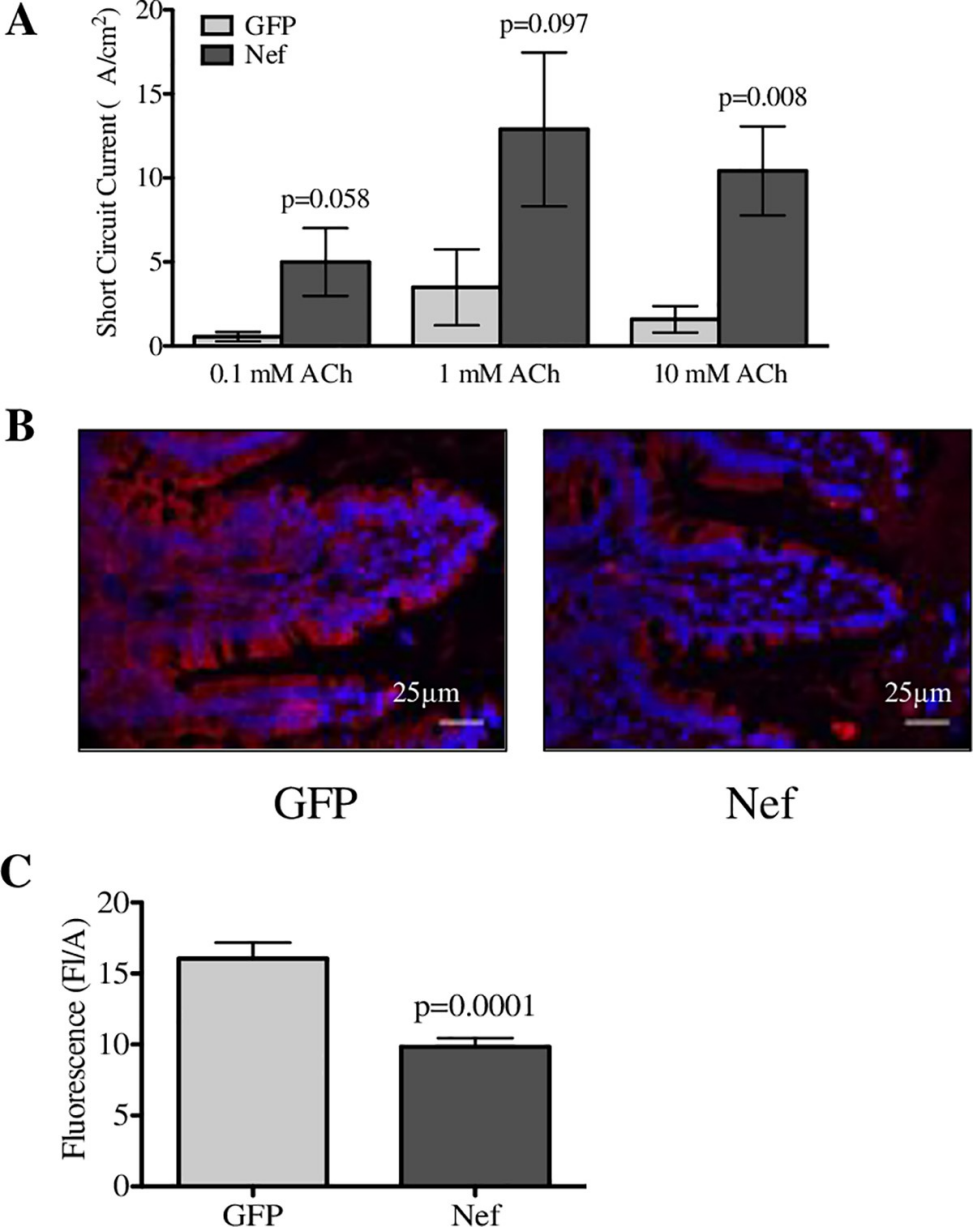
Nef expression in rat hippocampus increases intestinal ion transport and downregulates the tight junction protein occludin. (**A**) Ion transport was measured by mounting ileal tissue in Ussing chambers and recording changes in ion transport to different acetylcholine (ACh) concentrations applied to the serosal side. (**B**) Immunofluorescence of the tight junction protein occluding (in green) was assessed to determine intestinal barrier changes between the groups. (**C**) Analysis of the AFVE showed that ileal tissue from Nef-treated rats had significantly decreased occludin levels when compared to the GFP-treated rats. *N* = 7 to 9 rats per group. Scale bars indicate 25 μm. Student’s t-test was performed to determine differences between the Nef-treated group and the GFP-treated group (control). Data are expressed as means ± S.E.M.

Increased pro-inflammatory cytokine levels in blood and intestinal inflammation are associated with physiological changes in the GI tract [59, 60]. Therefore, we looked for changes in GI permeability. To assess changes in secretion, we measured epithelial ion transport by mounting ileal tissue, free from muscularis propria, in Ussing chambers. Changes in ion transport induced by increasing acetylcholine (ACh) concentrations were recorded using a voltmeter. The ileal tissue from Nef animals showed a trend of increased levels of ion transport starting at 0.1 mM acetylcholine that reached significance at 10 mM (Fig 5A), showing that Nef-treated rats had increased ileal secretion. Given that GI barrier permeability is controlled by tight junction proteins [61], we measured the tight junction protein occludin in the ileum by immunofluorescence intensity using ImageJ (Fig 5B). The AFVE from the ileal tissue of Nef animals was significantly decreased to about two-thirds of the staining of controls (Fig 5C), indicating that Nef-treated rats had less tight junctions and increased ileal barrier permeability.

### Hippocampal infusion of Nef-expressing astrocytes induces inflammation and increases permeability in the lung

**Fig 6:**
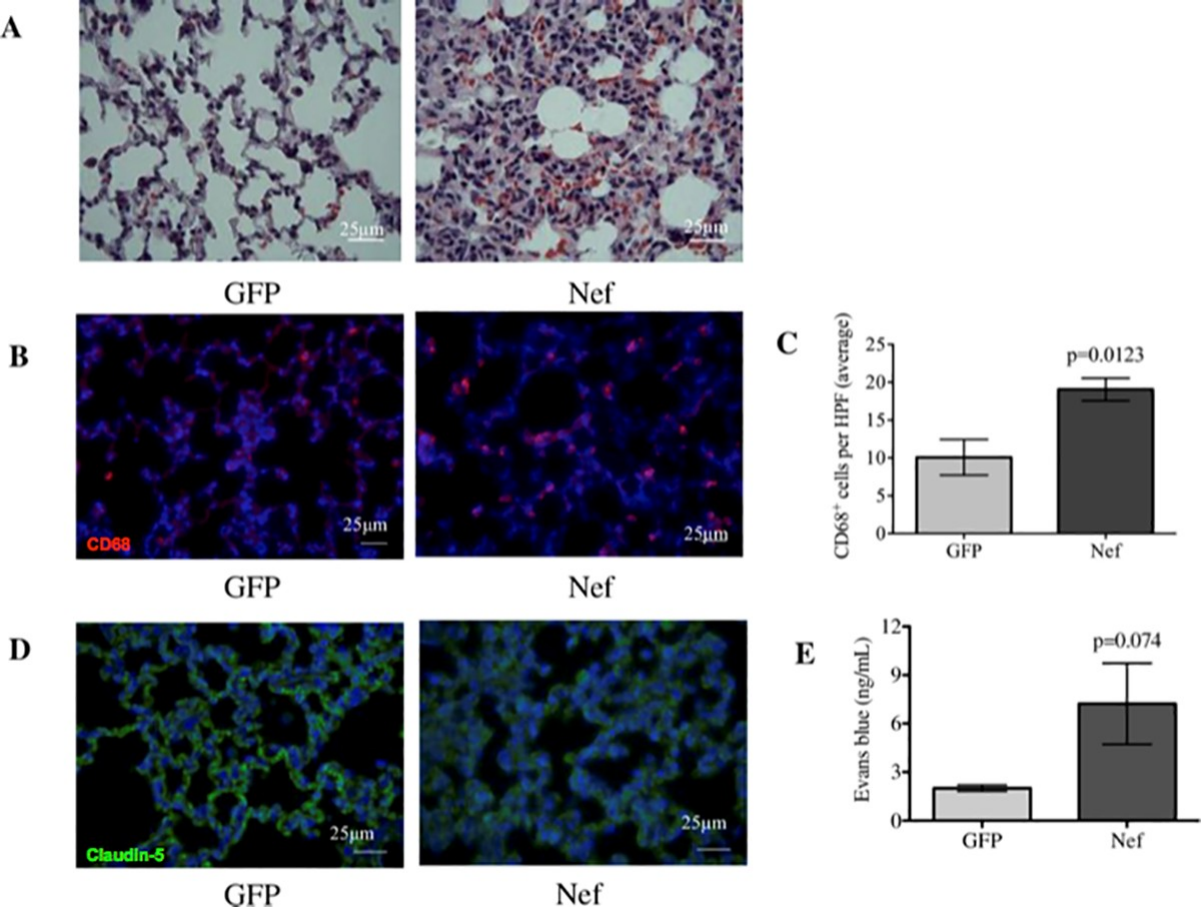
Nef expression in the rat hippocampus induces interstitial pneumonitis and increases macrophage infiltration and vascular permeability in the lung. **(A)** H&E staining showed that the lungs from GFP-treated rats appear normal, while the lungs from Nef-treated rats showed marked infiltration of immune cell and interstitial thickening. **(B)** Immunofluorescence for the macrophage marker CD68 in (red) while nuclei of the cells is represented in (blue) was assessed to compare macrophage infiltration between the groups. **(C)** Macrophages were quantified in three HPFs per rat. **(D)** Immunofluorescence of the tight junction protein claudin-5 in (green) while nuclei of the cells is represented in (blue) was assessed to determine tight junction alterations in the lungs. Nef-treated rats showed alterations in claudin-5 expression that were characterized by diffuse and dimmed staining compared to what was observed in the GFP-treated rats. **(E)** Changes in lung permeability were determined by measuring EB extravasation, which confirmed that Nef-treated rats had increased lung permeability compared to our control group. *N* = 4 to 6 rats per group. Scale bars indicate 25 μm. Student’s t-test was performed to determine differences between the Nef-treated group and the GFP-treated group (control). Data are expressed as means ± S.E.M.

The changes we observed in the brain [43] and ileum led us to examine effects on other organs by the infusion of Nef-expressing astrocytes into the hippocampus. We chose to examine the lung because of the crosstalk that has been reported to exist between pulmonary tissue, the gut [46, 47], and the brain [44, 45]. H&E staining showed that lung tissue from the Nef animals, but not from the GFP animals, had interstitial pneumonitis, characterized by marked lymphocyte, neutrophil (large, purple nucleus) and eosinophil (bright red cells) infiltration within the interstitium of the lung (Fig 6A). Immunofluorescent staining for CD68 revealed a significant increase in the prevalence of macrophages in the lung interstitium of Nef-treated rats in comparison to GFP-treated rats (Fig 6B and 6C). Claudin-5 expression was shown to be decreased in Nef animals, as determined by immunofluorescence (Fig 6D). In agreement with the findings mentioned above, increased extravasation of EB in the lung tissue of Nef-treated rats (Fig 6E) indicated enhanced vascular permeability in these rats when compared to GFP controls.

Thus, through a series of biochemical and functional experiments, we show that Nef expression by astrocytes generates inflammation and tissue pathology outside the brain. Specifically, ileal tissue has an expansion of lymphoid tissue, macrophage migration, loss of villus architecture, reduced junction protein expression and excessive secretory function. Lungs similarly are characterized by tissue changes reflecting inflammation, including interstitial pneumonitis, an influx of macrophage and eosinophils, and disrupted occludin protein and barrier function.

### Intracerebral ventricular infusion of IL-1β receptor antagonist prevents blood-brain-barrier disruption induced by Nef expression in the rat hippocampus

**Fig 7:**
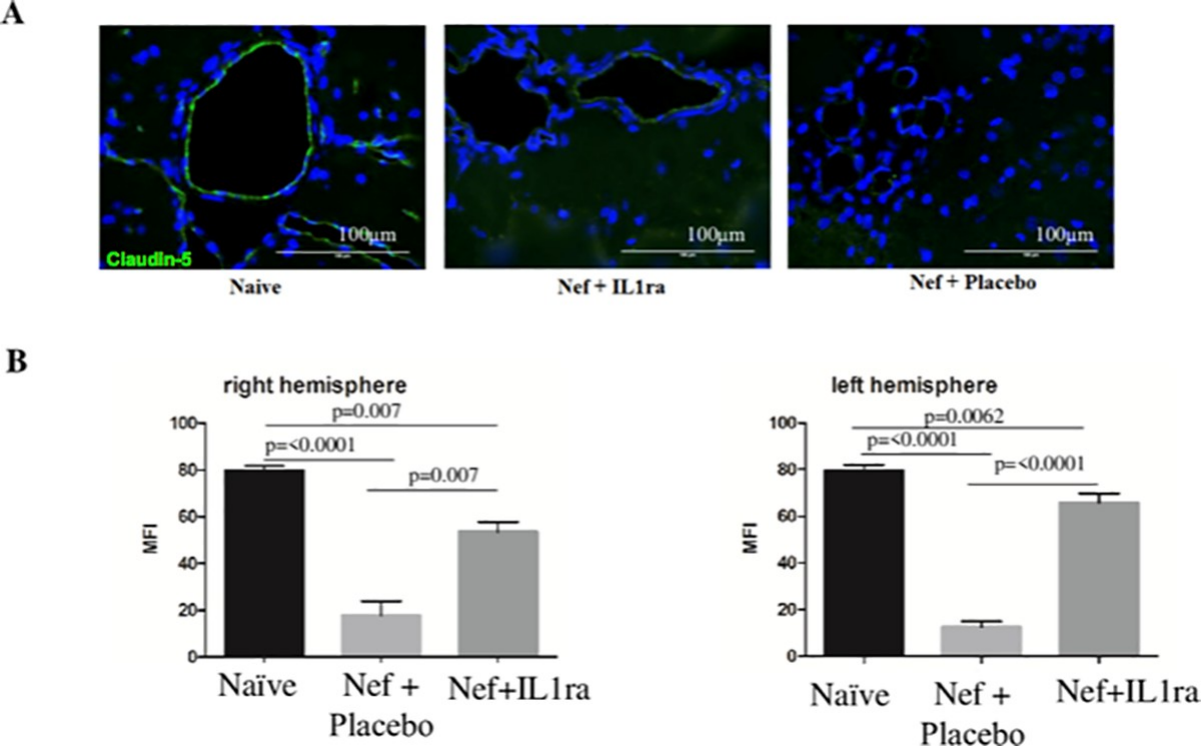
Intracerebral ventricular infusion of IL-1β receptor antagonist prevents blood-brain-barrier disruption induced by Nef expression in the rat hippocampus. **(A)** Immunofluorescence of claudin-5: the green represents the tight junction claudin-5, while the blue represents the nucleus of the cells. The results showed IL-1ra partially inhibits BBB disruption caused Nef. **(B)** Quantitative analysis of BBB tight junction claudin-5 showed that Nef decreased (by twofold) the expression of the tight junction compared to Nef with the IL-1β receptor antagonist in both hemispheres. *N* = 4 to 6 rats per group. One-way ANOVA (followed by Holm–Sidak’s multiple comparisons test) was used to determine the difference between naïve, Nef + placebo, and Nef + IL1-Ra. Data are expressed as means ± S.E.M.

Since we found a highly elevated levels of IL-1β and inflammation, we wanted to test the efficacy of blocking IL-1β to reduce the pathology observed in the brains, ileum, and lungs of our experimental animals. As Nef can induce inflammation that may alter BBB integrity, we infused IL1-Ra intracerebroventricularly to prevent the binding of IL-1β to its receptor. Immunofluorescence analysis of claudin-5 showed that IL1-Ra prevented the disruption of BBB tight junctions, with more uniform claudin-5 expression in the brains of Nef-IL1-Ra rats compared to those of Nef-placebo rats (Fig 7A). Quantitative analysis of the BBB protein claudin-5 demonstrated that Nef treatment alone decreased claudin-5 immunostaining, while Nef plus the IL1-Ra restored claudin-5 immunostaining to levels similar to those of the naive control (Fig 7B) in both hemispheres. These findings suggest that IL-1β could be mediating the inflammation caused by Nef in the different organs.

### Intracerebral ventricular infusion of IL1 receptor antagonist blocks systemic inflammatory response to hippocampal Nef expression

**Fig 8:**
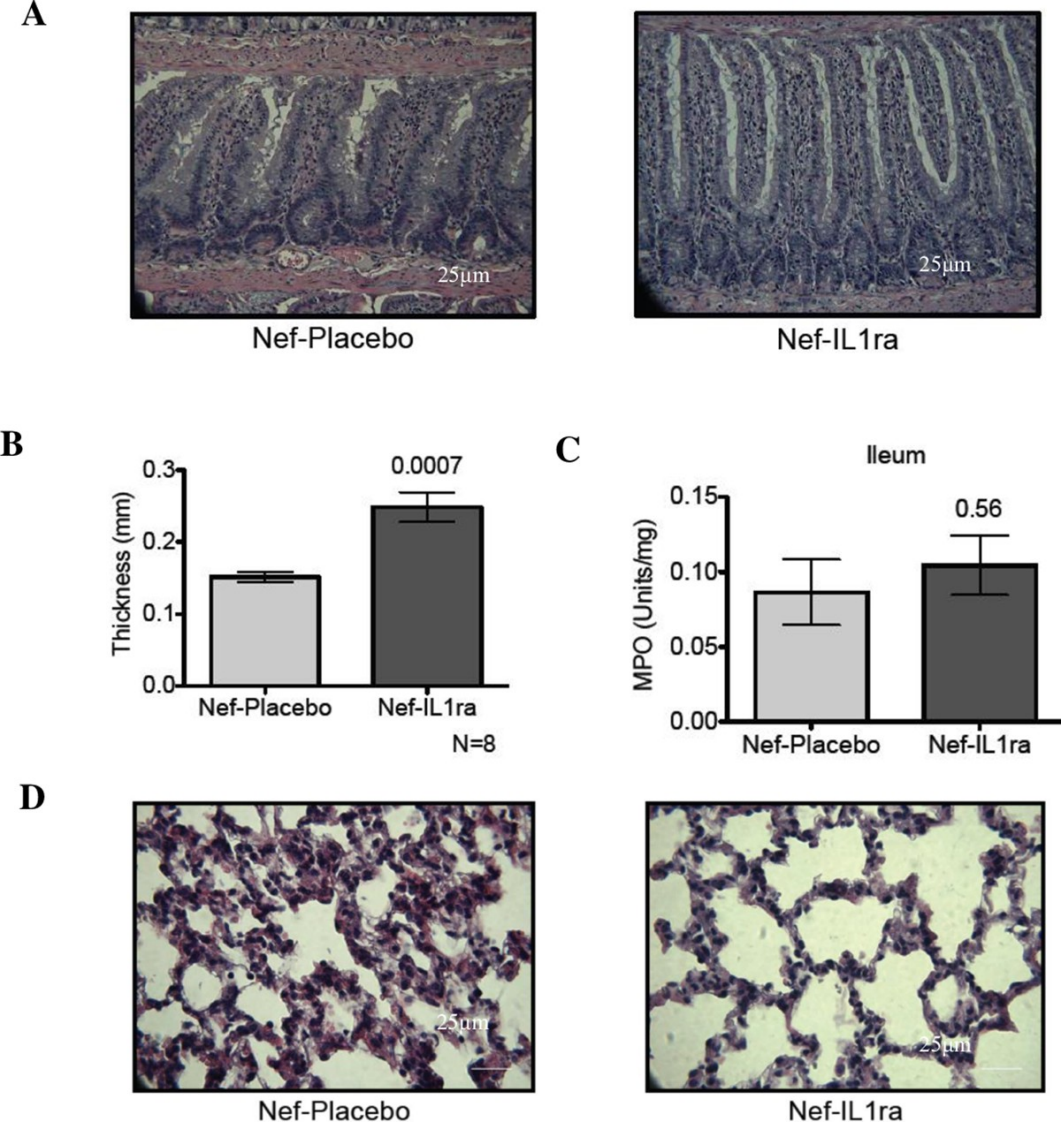
IL1ra administered intracerebroventricularly in rats expressing hippocampal Nef inhibits the development of ilium structural changes, tissue thickening, and lung interstitial pneumonitis. **(A)** H&E staining of ileal tissue (40X) showed that IL1ra treatment inhibits the shortening of the villi and the widening of the lamina propria. **(B)** Measurements of ileal tissue thickness showed that Nef-IL1ra rats had significantly increased thickness compared to the Nef-placebo rats. **(C)** Quantification of ileal MPO did not show inflammation differences between treatments. **(D)** H&E staining from lungs showed that intracerebral ventricular infusion treatment with IL1ra prevented the thickening of the interstitium and recruitment of eosinophils and neutrophils.

As anti-inflammatory treatment has been shown to reduce the peripheral response to injury in the CNS, we observed the prevention of most of the peripheral tissue inflammation caused by Nef in animals that were co-administered IL-1Ra. Fig 8 shows that ileum and lung tissue both show normal morphology in Nef-treated rats that also received an intracerebral ventricular infusion of IL1-Ra before and during treatment with Nef-transfected astrocytes. The ileal of Nef-treated animals that were intracerebroventricularly co-administered saline showed muscle-layer thinning and enlarged Peyer’s patches (Fig 8A), akin to what was observed when Nef, alone, was in place (Fig 3C). When Nef-treated rats also received IL-1Ra, the tissue’s appearance reverted to normal, and overall tissue thickness was increased significantly and near normal (Fig 8B); however, MPO levels remained elevated. Lung tissue histology also showed substantial improvement in IL1-Ra–treated rats (Fig 8D), with reduced thickening of the interstitium and decreased lymphocyte, eosinophil, and neutrophil infiltration.

## Discussion

Nef is an early HIV-1 protein that is expressed by latently infected cells, including astrocytes [62, 63]. Nef is highly neurotoxic and alone is sufficient to produce oxidative stress, axon/neurite degeneration, and lost neurotransmission, in vitro, as well as damage to the BBB [27, 64–67]. Prior work with this model and others has shown that HIV-1 Nef alone caused neurocognitive defects in rats [43, 68] and suggests a link with neurological disease in humans [69, 70]. Nef-treated rats exhibit macroscopic changes of the ileum and lung, which we aimed to define and characterize microscopically and physiologically in the present study. Herein we report that the infusion of Nef-expressing astrocytes into the rat hippocampus caused alterations within the brain—likely leading to neurocognitive deficits—and at several sites outside of the brain, including the ileum and lungs. Treatment with Nef caused pronounced morphological and inflammatory alterations in the ileum and compromised the integrity of this organ’s mucosal barrier. Ileal tissue from Nef-treated rats was thinner and demonstrated larger Peyer’s patches and smaller intestinal villi than did such tissue from the controls. The rats in the sample had augmented MPO activity, increased lymphocyte, eosinophil and macrophage infiltration, enhanced ion secretion in response to acetylcholine titration, and decreased epithelial expression of the tight junction protein occludin. The lungs of the Nef-treated rats revealed the infiltration of inflammatory cells which included lymphocytes, neutrophils, eosinophils, and macrophages within the interstitial spaces. Lungs demonstrated increased vascular permeability by EB and reduced occludin staining, reflecting these inflammatory changes. In addition to the previously described cognitive impairment and inflammatory changes in the brain [43], we observed an increase in BBB permeability, as evidenced by enhanced EB extravasation and decreased claudin-5 expression on vascular endothelial cells, in Nef-treated rats, compared to GFP-treated controls. Infusing astrocytes expressing HIV-1 Nef into the rat hippocampus increased the serum levels of IL-1β, thus providing evidence of the peripheral effects of infused Nef-expressing astrocytes. Finally, intracerebroventricular application of an IL-1 receptor antagonist was effective in partially restoring normal claudin-5 staining in the brain, GI and lung tissue morphology, but did not eliminate all tissue inflammation as demonstrated by continued elevation of ileal MPO activity.

Understanding the effects of Nef produced in the brain on peripheral organs is significant for the insight it provides on the role this protein plays in cART-treated HIV-1 infection and ensuing disease. Nef is expressed in different regions of the brain (e.g., prefrontal cortex, hippocampus) and by different cells (e.g., microglia, endothelial cells, astrocytes) with greater infection of astrocytes in cases of neuropathology [71]. Specifically, Nef RNA sequences and protein have been identified in hippocampal astrocytes in the post-mortem brain tissues of HIV patients [72–74] which is why we have used astrocytes to produce Nef in these studies. Research shows that Nef is neurotoxic [75–77]. In the lungs, HIV-1 Nef causes endothelial cell dysfunction and pulmonary hypertension [78–80]. Nef disrupts normal function of the intestinal mucosa [81].

Our findings suggest a role for HIV-1 Nef expressed by astrocytes in disease processes that affect the GI tracts of HIV-infected patients. HIV infection leads to the recruitment of T-cells and other leukocytes to the gut-associated lymphoid tissue (GALT), where CCR5^+^CD4^+^ T-cells and the GALT are depleted rapidly [82–84]. In contrast to the GALT reduction observed in HIV and SIV infection, our Nef-treated rats experienced pronounced GALT expansion, demonstrated by the increase in size and number of Peyer’s patches. HIV-1 Nef has been reported to activate macrophages and to stimulate their migration to the gut [85, 86]. Nef can decrease the migratory potential of T-cells [87–89]. Therefore, once uninfected T-cells have been recruited to the lamina propria, Nef can potentially trap these T-cells in the gut, facilitating their infection with the virus. HIV-1 Nef could also be a factor that drives HIV enteropathy [81], which is characterized by chronic malabsorption, malnutrition, and diarrhea secondary to intestinal damage and inflammation [90, 91]. In our model, the expression of Nef, alone, albeit in astrocytes in the hippocampus, caused villous atrophy and inflammatory cell infiltration, enhanced epithelial secretion, and compromised barrier integrity, all of which could contribute to the development of diarrhea.

Our model shows that Nef can enhance the secretion of IL-1β suggesting a role in the pathology induced by the Nef protein. Therefore, to test for such a role, we antagonized its action by administering the recombinant form of the IL-1β receptor antagonist. Our results demonstrated the restoration of BBB integrity in the brain. Peripheral tissues showed healthy ileal architecture, decreased neutrophil infiltration in the ileum, and reduced infiltration of eosinophils and neutrophils in the lungs. These results suggested that IL-1β plays role in mediating peripheral inflammation.

Although, we have observed several peripheral effects resulting from the infusion of Nef-expressing astrocytes into the hippocampus, the underlying mechanisms responsible for these changes remain unknown. Our findings suggest that inflammatory mediators, such as IL-1β, are important in linking the neurotoxic effects of Nef to changes in peripheral tissues. Nef can activate macrophages promoting the secretion of proinflammatory cytokines [85], which can spread across the brain and induce inflammatory processes distant from where they were secreted [92]. IL-1β can alter tight junction proteins and provoke BBB disruption, which may promote the entry of immune cells and pathogenic microbes into the brain [93, 94] as well as leak proinflammatory cytokines from the brain into the circulatory system. Astrocytes expressing Nef and activating microglia/macrophages are known to secrete IL-1β. Interestingly, in traumatic brain injury (TBI) which shares some pathological sequalae to our model, IL-1β is also upregulated in the brain as early as three hours after injury [35]. TBI patients may also present BBB disruption, systemic inflammation, pneumonia, increased intestinal secretion, blunting, and architectural changes of intestinal villi [35, 40, 44]. Similarly, neuroinflammation induced by Nef may be involved in BBB integrity alterations and permeability, systemic inflammation, and peripheral pathologies.

The contribution of astrocytes in early HIV infection is yet to be clearly identified in comparison to cells such as macrophages and microglia which are universally held as a major site of HIV infection and replication in the brain. Indeed, the infection of astrocytes remains controversial (discussed in refs [6, 95, 96]) as the nature of entry is unclear due to the lack of cell surface CD4 receptor expression [97, 98]; entry of the virus could occur via endocytosis[99]. Astrocytes are also reported to trap viral proteins secreted by microglia [95]. Astrocyte infection results in a latent, non-productive infection characterized by early, non-structural protein production [12, 100]. Studies demonstrate Nef and Tat expression in patients with HAND [101–103]. Our findings in a rat model have clear limitations in translation to human HIV infection. Still, the present study demonstrates that astrocytes with productive expression of the Nef protein could contribute to both local and systemic inflammatory processes.

In conclusion, our work demonstrated that Nef expression is neurotoxic, neuroinflammatory, and supports our findings relevant to learning impairment [43]. We found increased IL-1β levels present with reduced junction protein expression in brain, ileum, and lung, which is consistent with existing literature. We reported evidence of functional disruption in barriers in each of these tissues by EB permeability, as well as tissue inflammation in ileum and lung by infiltration of innate immune cells and tissue disruption. Ileal tissues showed elevated secretory responses to acetylcholine stimulation as a consequence of inflammation driven by Nef expression from astrocytes. Many of these findings were reversed or reduced in severity by application of IL1ra in brain, suggesting IL-1 β helps in mediating the effects of Nef. These findings highlight the impact of viral protein expression, which is not a specific target of current antiretroviral medications, as a contributing factor to the multi-pathology that affects HIV-positive patients and argue for early viral protein expression from latent virus as a therapeutic target.

## Supporting information

S1 File Supporting information file. (XLSX)

## References

[1] Gupta RK, Wainberg MA, Brun-Vezinet F, Gatell JM, Albert J, Sonnerborg A, et al Oral antiretroviral drugs as public health tools for HIV prevention: global implications for adherence, drug resistance, and the success of HIV treatment programs. J Infect Dis. 2013;207 Suppl 2:S101–6. Epub 2013/05/25. 10.1093/infdis/jit108 23687287 PMC3708737

[2] Kaul M, Zheng J, Okamoto S, Gendelman HE, Lipton SA. HIV-1 infection and AIDS: consequences for the central nervous system. Cell Death Differ. 2005;12 Suppl 1:878–92. Epub 2005/04/16. 10.1038/sj.cdd.4401623 . 15832177

[3] Churchill MJ, Wesselingh SL, Cowley D, Pardo CA, McArthur JC, Brew BJ, et al Extensive astrocyte infection is prominent in human immunodeficiency virus-associated dementia. Annals of neurology. 2009;66(2):253–8. Epub 2009/09/11. 10.1002/ana.21697 . 19743454

[4] Huang Z, Nair M. A CRISPR/Cas9 guidance RNA screen platform for HIV provirus disruption and HIV/AIDS gene therapy in astrocytes. Scientific Reports. 2017;7(1):5955 10.1038/s41598-017-06269-x 28729655 PMC5519727

[5] Barat C, Proust A, Deshiere A, Leboeuf M, Drouin J, Tremblay MJ. Astrocytes sustain long-term productive HIV-1 infection without establishment of reactivable viral latency. Glia. 2018;66(7):1363–81. 10.1002/glia.23310 29464785

[6] Al-Harthi L, Nath A. Letter to Editor. Journal of neuroimmune pharmacology: the official journal of the Society on NeuroImmune Pharmacology. 2019;14(1):6 Epub 2018/12/14. 10.1007/s11481-018-09827-w . 30542907

[7] Henderson LJ, Narasipura SD, Adarichev V, Kashanchi F, Al-Harthi L. Identification of Novel T Cell Factor 4 (TCF-4) Binding Sites on the HIV Long Terminal Repeat Which Associate with TCF-4, β-Catenin, and SMAR1 To Repress HIV Transcription. Journal of Virology. 2012;86(17):9495–503. 10.1128/JVI.00486-12 22674979 PMC3416155

[8] Narasipura SD, Kim S, Al-Harthi L. Epigenetic Regulation of HIV-1 Latency in Astrocytes. Journal of Virology. 2014;88(5):3031–8. 10.1128/JVI.03333-13 24352441 PMC3958059

[9] Li GH, Henderson L, Nath A. Astrocytes as an HIV Reservoir: Mechanism of HIV Infection. Curr HIV Res. 2016;14(5):373–81. Epub 2016/10/11. 10.2174/1570162x14666161006121455 . 27719663 PMC11345863

[10] Prevedel L, Ruel N, Castellano P, Smith C, Malik S, Villeux C, et al Identification, Localization, and Quantification of HIV Reservoirs Using Microscopy. Current protocols in cell biology. 2019;82(1):e64 Epub 2018/09/29. 10.1002/cpcb.64 30265439 PMC6386609

[11] Rao VR, Eugenin EA, Prasad VR. Evaluating the Role of Viral Proteins in HIV-Mediated Neurotoxicity Using Primary Human Neuronal Cultures. Methods in molecular biology (Clifton, NJ). 2016;1354:367–76. Epub 2015/12/31. 10.1007/978-1-4939-3046-3_25 26714725 PMC5050920

[12] Eugenin EA, Clements JE, Zink MC, Berman JW. Human immunodeficiency virus infection of human astrocytes disrupts blood-brain barrier integrity by a gap junction-dependent mechanism. The Journal of neuroscience: the official journal of the Society for Neuroscience. 2011;31(26):9456–65. Epub 2011/07/01. 10.1523/jneurosci.1460-11.2011 21715610 PMC3132881

[13] Luo X, He JJ. Cell-cell contact viral transfer contributes to HIV infection and persistence in astrocytes. Journal of neurovirology. 2015;21(1):66–80. Epub 2014/12/19. 10.1007/s13365-014-0304-0 . 25522787 PMC4861053

[14] Li GH, Anderson C, Jaeger L, Do T, Major EO, Nath A. Cell-to-cell contact facilitates HIV transmission from lymphocytes to astrocytes via CXCR4. Aids. 2015;29(7):755–66. Epub 2015/05/20. 10.1097/QAD.0000000000000605 25985398 PMC4438861

[15] Asahchop EL, Meziane O, Mamik MK, Chan WF, Branton WG, Resch L, et al Reduced antiretroviral drug efficacy and concentration in HIV-infected microglia contributes to viral persistence in brain. Retrovirology. 2017;14(1):47 Epub 2017/10/19. 10.1186/s12977-017-0370-5 29037245 PMC5644262

[16] Khan MB, Lang MJ, Huang M-B, Raymond A, Bond VC, Shiramizu B, et al Nef exosomes isolated from the plasma of individuals with HIV-associated dementia (HAD) can induce Aβ1–42 secretion in SH-SY5Y neural cells. Journal of neurovirology. 2016;22(2):179–90. 10.1007/s13365-015-0383-6 26407718 PMC4783240

[17] Raymond AD, Diaz P, Chevelon S, Agudelo M, Yndart-Arias A, Ding H, et al Microglia-derived HIV Nef+ exosome impairment of the blood-brain barrier is treatable by nanomedicine-based delivery of Nef peptides. Journal of neurovirology. 2016;22(2):129–39. Epub 2015/12/04. 10.1007/s13365-015-0397-0 . 26631079

[18] Xu W, Santini PA, Sullivan JS, He B, Shan M, Ball SC, et al HIV-1 evades virus-specific IgG2 and IgA responses by targeting systemic and intestinal B cells via long-range intercellular conduits. Nature immunology. 2009;10:1008–17. 10.1038/ni.1753 . 19648924 PMC2784687

[19] Uhl J, Gujarathi S, Waheed AA, Gordon A, Freed EO, Gousset K. Myosin-X is essential to the intercellular spread of HIV-1 Nef through tunneling nanotubes. Journal of cell communication and signaling. 2018 Epub 2018/11/18. 10.1007/s12079-018-0493-z . 30443895 PMC6498342

[20] Wang T, Green LA, Gupta SK, Kim C, Wang L, Almodovar S, et al Transfer of intracellular HIV Nef to endothelium causes endothelial dysfunction. PLoS ONE. 2014;9(3):e91063 Epub 2014/03/13. 10.1371/journal.pone.0091063 24608713 PMC3946685

[21] Ferdin J, Goričar K, Dolžan V, Plemenitaš A, Martin JN, Peterlin BM, et al Viral protein Nef is detected in plasma of half of HIV-infected adults with undetectable plasma HIV RNA. PloS one. 2018;13(1):e0191613 10.1371/journal.pone.0191613 29364927 PMC5783402

[22] Chaudhuri R, Lindwasser OW, Smith WJ, Hurley JH, Bonifacino JS. Downregulation of CD4 by human immunodeficiency virus type 1 Nef is dependent on clathrin and involves direct interaction of Nef with the AP2 clathrin adaptor. Journal of virology. 2007;81:3877–90. 10.1128/JVI.02725-06 . 17267500 PMC1866153

[23] Garcia JV, Miller AD. Serine phosphorylation-independent downregulation of cell-surface CD4 by nef. Nature. 1991;350:508–11. 10.1038/350508a0 . 2014052

[24] Le Gall S, Erdtmann L, Benichou S, Berlioz-Torrent C, Liu L, Benarous R, et al Nef interacts with the mu subunit of clathrin adaptor complexes and reveals a cryptic sorting signal in MHC I molecules. Immunity. 1998;8:483–95. 10.1016/s1074-7613(00)80553-1 . 9586638

[25] Schwartz O, Maréchal V, Le Gall S, Lemonnier F, Heard JM. Endocytosis of major histocompatibility complex class I molecules is induced by the HIV-1 Nef protein. Nature medicine. 1996;2:338–42. 10.1038/nm0396-338 . 8612235

[26] Pužar Dominkuš P, Ferdin J, Plemenitaš A, Peterlin BM, Lenassi M. Nef is secreted in exosomes from Nef.GFP-expressing and HIV-1-infected human astrocytes. Journal of neurovirology. 2017;23(5):713–24. Epub 2017/08/02. 10.1007/s13365-017-0552-x 28762184 PMC6010353

[27] Sami Saribas A, Cicalese S, Ahooyi TM, Khalili K, Amini S, Sariyer IK. HIV-1 Nef is released in extracellular vesicles derived from astrocytes: evidence for Nef-mediated neurotoxicity. Cell Death Dis. 2017;8:e2542 10.1038/cddis.2016.467 28079886 PMC5386374

[28] Liu X, Shah A, Gangwani MR, Silverstein PS, Fu M, Kumar A. HIV-1 Nef induces CCL5 production in astrocytes through p38-MAPK and PI3K/Akt pathway and utilizes NF-kB, CEBP and AP-1 transcription factors. Scientific reports. 2014;4:4450 Epub 2014/03/25. 10.1038/srep04450 24658403 PMC3963078

[29] Nitkiewicz J, Borjabad A, Morgello S, Murray J, Chao W, Emdad L, et al HIV induces expression of complement component C3 in astrocytes by NF-κB-dependent activation of interleukin-6 synthesis. J Neuroinflammation. 2017;14(1):23 Epub 2017/01/27. 10.1186/s12974-017-0794-9 28122624 PMC5267445

[30] Gallo P, Frei K, Rordorf C, Lazdins J, Tavolato B, Fontana A. Human immunodeficiency virus type 1 (HIV-1) infection of the central nervous system: an evaluation of cytokines in cerebrospinal fluid. Journal of neuroimmunology. 1989;23:109–16. 10.1016/0165-5728(89)90029-5 . 2656753

[31] Lau LT, Yu AC-H. Astrocytes Produce and Release Interleukin-1, Interleukin-6, Tumor Necrosis Factor Alpha and Interferon-Gamma Following Traumatic and Metabolic Injury. Journal of Neurotrauma. 2001;18(3):351–9. 10.1089/08977150151071035 . 11284554

[32] Parnet P, Kelley KW, Bluthe RM, Dantzer R. Expression and regulation of interleukin-1 receptors in the brain. Role in cytokines-induced sickness behavior. J Neuroimmunol. 2002;125(1–2):5–14. Epub 2002/04/19. 10.1016/s0165-5728(02)00022-x . 11960635

[33] Lampa J, Westman M, Kadetoff D, Agreus AN, Le Maitre E, Gillis-Haegerstrand C, et al Peripheral inflammatory disease associated with centrally activated IL-1 system in humans and mice. Proc Natl Acad Sci U S A. 2012;109(31):12728–33. Epub 2012/07/18. 10.1073/pnas.1118748109 22802629 PMC3411968

[34] Schenk M, Fabri M, Krutzik SR, Lee DJ, Vu DM, Sieling PA, et al Interleukin-1β triggers the differentiation of macrophages with enhanced capacity to present mycobacterial antigen to T cells. Immunology. 2014;141(2):174–80. Epub 2013/09/17. 10.1111/imm.12167 24032597 PMC3904238

[35] Lu J, Goh SJ, Tng PYL, Deng YY, Ling E-A, Moochhala S. Systemic inflammatory response following acute traumatic brain injury. Frontiers in bioscience (Landmark edition). 2009;14:3795–813. 10.2741/3489 . 19273311

[36] Eng LF, Ghirnikar RS. GFAP and astrogliosis. Brain Pathol. 1994;4(3):229–37. 10.1111/j.1750-3639.1994.tb00838.x 7952264

[37] Lu J, Goh SJ, Tng PY, Deng YY, Ling EA, Moochhala S. Systemic inflammatory response following acute traumatic brain injury. Frontiers in bioscience. 2009;14:3795–813. Epub 2009/03/11. 10.2741/3489 . 19273311

[38] Sato M, Chang E, Igarashi T, Noble LJ. Neuronal injury and loss after traumatic brain injury: time course and regional variability. Brain Res. 2001;917(1):45–54. Epub 2001/10/17. 10.1016/s0006-8993(01)02905-5 . 11602228

[39] Akay C, Lindl KA, Shyam N, Nabet B, Goenaga-Vazquez Y, Ruzbarsky J, et al Activation status of integrated stress response pathways in neurons and astrocytes of HAND cortex. Neuropathology and Applied Neurobiology. 2011:no-no. 10.1111/j.1365-2990.2011.01215.x 21883374 PMC3708539

[40] Bansal V, Costantini T, Kroll L, Peterson C, Loomis W, Eliceiri B, et al Traumatic brain injury and intestinal dysfunction: uncovering the neuro-enteric axis. Journal of neurotrauma. 2009;26:1353–9. 10.1089/neu.2008-0858 . 19344293 PMC2989839

[41] Hang C-H, Shi J-X, Li J-S, Wu W, Yin H-X. Alterations of intestinal mucosa structure and barrier function following traumatic brain injury in rats. World journal of gastroenterology. 2003;9:2776–81. 10.3748/wjg.v9.i12.2776 . 14669332 PMC4612051

[42] Kao CH, ChangLai SP, Chieng PU, Yen TC. Gastric emptying in head-injured patients. The American journal of gastroenterology. 1998;93:1108–12. 10.1111/j.1572-0241.1998.00338.x . 9672339

[43] Chompre G, Cruz E, Maldonado L, Rivera-Amill V, Porter JT, Noel RJ. Astrocytic expression of HIV-1 Nef impairs spatial and recognition memory. Neurobiology of disease. 2013;49:128–36. 10.1016/j.nbd.2012.08.007 . 22926191 PMC3530662

[44] Mascia L. Acute lung injury in patients with severe brain injury: a double hit model. Neurocritical care. 2009;11:417–26. 10.1007/s12028-009-9242-8 . 19548120

[45] Pelosi P, Rocco PR. The lung and the brain: a dangerous cross-talk. Crit Care. 2011;15(3):168 10.1186/cc10259 21722336 PMC3219008

[46] Ichinohe T, Pang IK, Kumamoto Y, Peaper DR, Ho JH, Murray TS, et al Microbiota regulates immune defense against respiratory tract influenza A virus infection. Proceedings of the National Academy of Sciences of the United States of America. 2011;108:5354–9. 10.1073/pnas.1019378108 . 21402903 PMC3069176

[47] Keely S, Talley NJ, Hansbro PM. Pulmonary-intestinal cross-talk in mucosal inflammatory disease. Mucosal immunology. 2012;5:7–18. 10.1038/mi.2011.55 . 22089028 PMC3243663

[48] Crothers K, Thompson BW, Burkhardt K, Morris A, Flores SC, Diaz PT, et al HIV-associated lung infections and complications in the era of combination antiretroviral therapy. Proceedings of the American Thoracic Society. 2011;8:275–81. 10.1513/pats.201009-059WR . 21653528 PMC3132785

[49] Plata-Salaman CR, Ffrench-Mullen JM. Intracerebroventricular administration of a specific IL-1 receptor antagonist blocks food and water intake suppression induced by interleukin-1 beta. Physiology & behavior. 1992;51(6):1277–9. Epub 1992/06/01. 10.1016/0031-9384(92)90321-r . 1386466

[50] Isidro RA, Isidro AA, Cruz ML, Hernandez S, Appleyard CB. Double immunofluorescent staining of rat macrophages in formalin-fixed paraffin-embedded tissue using two monoclonal mouse antibodies. Histochemistry and Cell Biology. 2015;144(6):613–21. 10.1007/s00418-015-1364-9 . 26403093 PMC4758119

[51] Gavet O, Pines J. Activation of cyclin B1-Cdk1 synchronizes events in the nucleus and the cytoplasm at mitosis. J Cell Biol. 2010;189(2):247–59. 10.1083/jcb.200909144 20404109 PMC2856909

[52] Appleyard CB, Alvarez A, Percy WH. Temporal changes in colonic vascular architecture and inflammatory mediator levels in animal models of colitis. Dig Dis Sci. 2002;47(9):2007–14. 10.1023/a:1019660526241 . 12353846

[53] Appleyard CB, Morales M, Percy WH. Regional variations in neurokinin receptor subtype contributions to muscularis mucosae and epithelial function in rat colon. Dig Dis Sci. 2006;51(3):506–16. 10.1007/s10620-006-3163-6 . 16614960

[54] Li H, Sheppard DN, Hug MJ. Transepithelial electrical measurements with the Ussing chamber. J Cyst Fibros. 2004;3 Suppl 2:123–6. 10.1016/j.jcf.2004.05.026 . 15463943

[55] Saria A, Lundberg JM. Evans blue fluorescence: quantitative and morphological evaluation of vascular permeability in animal tissues. J Neurosci Methods. 1983;8(1):41–9. 10.1016/0165-0270(83)90050-x . 6876872

[56] de Vries HE, Kuiper J, de Boer AG, Van Berkel TJ, Breimer DD. The blood-brain barrier in neuroinflammatory diseases. Pharmacological reviews. 1997;49:143–55. . 9228664

[57] Elices MJ. Neuroinflammatory diseases and the importance of a healthy blood-brain barrier. Current opinion in investigational drugs (London, England: 2000). 2008;9:1149–50. . 18951292

[58] Lepe-Zuniga JL, Mansell PW, Hersh EM. Idiopathic production of interleukin-1 in acquired immune deficiency syndrome. Journal of clinical microbiology. 1987;25:1695–700. . 3498739 10.1128/jcm.25.9.1695-1700.1987 PMC269310

[59] Machowska A, Brzozowski T, Sliwowski Z, Pawlik M, Konturek PC, Pajdo R, et al Gastric secretion, proinflammatory cytokines and epidermal growth factor (EGF) in the delayed healing of lingual and gastric ulcerations by testosterone. Inflammopharmacology. 2008;16(1):40–7. 10.1007/s10787-007-1600-6 . 18046513

[60] Spindler-Vesel A, Wraber B, Vovk I, Kompan L. Intestinal permeability and cytokine inflammatory response in multiply injured patients. J Interferon Cytokine Res. 2006;26(10):771–6. 10.1089/jir.2006.26.771 . 17032171

[61] Gasbarrini G, Montalto M. Structure and function of tight junctions. Role in intestinal barrier. Italian journal of gastroenterology and hepatology. 31:481–8. . 10575567

[62] Brack-Werner R. Astrocytes: HIV cellular reservoirs and important participants in neuropathogenesis. Aids. 1999;13(1):1–22. Epub 1999/04/20. 10.1097/00002030-199901140-00003 . 10207540

[63] Brack-Werner R, Kleinschmidt A, Ludvigsen A, Mellert W, Neumann M, Herrmann R, et al Infection of human brain cells by HIV-1: restricted virus production in chronically infected human glial cell lines. Aids. 1992;6(3):273–85. Epub 1992/03/01. . 1373627

[64] Acheampong EA, Parveen Z, Muthoga LW, Kalayeh M, Mukhtar M, Pomerantz RJ. Human Immunodeficiency virus type 1 Nef potently induces apoptosis in primary human brain microvascular endothelial cells via the activation of caspases. J Virol. 2005;79(7):4257–69. 10.1128/JVI.79.7.4257-4269.2005 15767427 PMC1061575

[65] Sporer B, Koedel U, Paul R, Kohleisen B, Erfle V, Fontana A, et al Human immunodeficiency virus type-1 Nef protein induces blood-brain barrier disruption in the rat: role of matrix metalloproteinase-9. J Neuroimmunol. 2000;102(2):125–30. 10.1016/s0165-5728(99)00170-8 10636480

[66] Van Marle G, Henry S, Todoruk T, Sullivan A, Silva C, Rourke SB, et al Human immunodeficiency virus type 1 Nef protein mediates neural cell death: a neurotoxic role for IP-10. Virology. 2004;329(2):302–18. 10.1016/j.virol.2004.08.024 15518810

[67] Acharjee S, Branton WG, Vivithanaporn P, Maingat F, Paul AM, Dickie P, et al HIV-1 Nef expression in microglia disrupts dopaminergic and immune functions with associated mania-like behaviors. Brain, behavior, and immunity. 2014;40:74–84. 10.1016/j.bbi.2014.02.016 24607605

[68] Mordelet E, Kissa K, Cressant A, Gray F, Ozden S, Vidal C, et al Histopathological and cognitive defects induced by Nef in the brain. Faseb J. 2004;18(15):1851–61. 10.1096/fj.04-2308com 15576488

[69] Lamers SL, Fogel GB, Liu ES, Barbier AE, Rodriguez CW, Singer EJ, et al Brain-specific HIV Nef identified in multiple patients with neurological disease. Journal of neurovirology. 2018;24(1):1–15. Epub 2017/10/25. 10.1007/s13365-017-0586-0 29063512 PMC5792318

[70] Lamers SL, Poon AFY, McGrath MS. HIV-1 Nef Protein Structures Associated with Brain Infection and Dementia Pathogenesis. PLoS ONE. 2011;6(2):e16659 10.1371/journal.pone.0016659 21347424 PMC3036659

[71] Churchill MJ, Wesselingh SL, Cowley D, Pardo CA, McArthur JC, Brew BJ, et al Extensive astrocyte infection is prominent in human immunodeficiency virus-associated dementia. Annals of neurology. 2009;66:253–8. 10.1002/ana.21697 . 19743454

[72] Conant K, Tornatore C, Atwood W, Meyers K, Traub R, Major EO. In vivo and in vitro infection of the astrocyte by HIV-1. Advances in neuroimmunology. 1994;4:287–9. 10.1016/s0960-5428(06)80269-x . 7874397

[73] Ranki A, Nyberg M, Ovod V, Haltia M, Elovaara I, Raininko R, et al Abundant expression of HIV Nef and Rev proteins in brain astrocytes in vivo is associated with dementia. AIDS (London, England). 1995;9:1001–8. 10.1097/00002030-199509000-00004 . 8527071

[74] Torres-Muñoz J, Stockton P, Tacoronte N, Roberts B, Maronpot RR, Petito CK. Detection of HIV-1 gene sequences in hippocampal neurons isolated from postmortem AIDS brains by laser capture microdissection. Journal of neuropathology and experimental neurology. 2001;60:885–92. 10.1093/jnen/60.9.885 . 11556545

[75] Speth C, Schabetsberger T, Mohsenipour I, Stöckl G, Würzner R, Stoiber H, et al Mechanism of human immunodeficiency virus-induced complement expression in astrocytes and neurons. Journal of virology. 2002;76:3179–88. 10.1128/JVI.76.7.3179-3188.2002 . 11884542 PMC136041

[76] Trillo-Pazos G, McFarlane-Abdulla E, Campbell IC, Pilkington GJ, Everall IP. Recombinant nef HIV-IIIB protein is toxic to human neurons in culture. Brain research. 2000;864:315–26. 10.1016/s0006-8993(00)02213-7 . 10802040

[77] Werner T, Ferroni S, Saermark T, Brack-Werner R, Banati RB, Mager R, et al HIV-1 Nef protein exhibits structural and functional similarity to scorpion peptides interacting with K+ channels. AIDS (London, England). 1991;5:1301–8. 10.1097/00002030-199111000-00003 . 1768378

[78] Almodovar S, Hsue PY, Morelli J, Huang L, Flores SC. Pathogenesis of HIV-associated pulmonary hypertension: potential role of HIV-1 Nef. Proceedings of the American Thoracic Society. 2011;8:308–12. 10.1513/pats.201006-046WR . 21653533 PMC3132790

[79] Marecki JC, Cool CD, Parr JE, Beckey VE, Luciw PA, Tarantal AF, et al HIV-1 Nef is associated with complex pulmonary vascular lesions in SHIV-nef-infected macaques. American journal of respiratory and critical care medicine. 2006;174:437–45. 10.1164/rccm.200601-005OC . 16728715 PMC2648120

[80] Sehgal PB, Mukhopadhyay S, Patel K, Xu F, Almodóvar S, Tuder RM, et al Golgi dysfunction is a common feature in idiopathic human pulmonary hypertension and vascular lesions in SHIV-nef-infected macaques. American journal of physiology Lung cellular and molecular physiology. 2009;297:L729–37. 10.1152/ajplung.00087.2009 . 19648286 PMC2770782

[81] Quaranta MG, Vincentini O, Felli C, Spadaro F, Silano M, Moricoli D, et al Exogenous HIV-1 Nef Upsets the IFN-γ-Induced Impairment of Human Intestinal Epithelial Integrity. PLoS ONE. 2011;6(8):e23442 10.1371/journal.pone.0023442 21858117 PMC3152569

[82] Brenchley JM, Schacker TW, Ruff LE, Price DA, Taylor JH, Beilman GJ, et al CD4+ T cell depletion during all stages of HIV disease occurs predominantly in the gastrointestinal tract. The Journal of experimental medicine. 2004;200:749–59. 10.1084/jem.20040874 . 15365096 PMC2211962

[83] Mehandru S, Poles MA, Tenner-Racz K, Horowitz A, Hurley A, Hogan C, et al Primary HIV-1 infection is associated with preferential depletion of CD4+ T lymphocytes from effector sites in the gastrointestinal tract. The Journal of experimental medicine. 2004;200:761–70. 10.1084/jem.20041196 . 15365095 PMC2211967

[84] Sankaran S, George MD, Reay E, Guadalupe M, Flamm J, Prindiville T, et al Rapid onset of intestinal epithelial barrier dysfunction in primary human immunodeficiency virus infection is driven by an imbalance between immune response and mucosal repair and regeneration. Journal of virology. 2008;82:538–45. 10.1128/JVI.01449-07 . 17959677 PMC2224350

[85] Swingler S, Mann A, Jacqué J, Brichacek B, Sasseville VG, Williams K, et al HIV-1 Nef mediates lymphocyte chemotaxis and activation by infected macrophages. Nature medicine. 1999;5:997–103. 10.1038/12433 . 10470075 PMC9513713

[86] Vérollet C, Souriant S, Bonnaud E, Jolicoeur P, Raynaud-Messina B, Kinnaer C, et al HIV-1 reprograms the migration of macrophages. Blood. 2015;125:1611–22. 10.1182/blood-2014-08-596775 . 25527710

[87] Choe EY, Schoenberger ES, Groopman JE, Park I-W. HIV Nef inhibits T cell migration. The Journal of biological chemistry. 2002;277:46079–84. 10.1074/jbc.M204698200 . 12354773

[88] Nobile C, Rudnicka D, Hasan M, Aulner N, Porrot F, Machu C, et al HIV-1 Nef inhibits ruffles, induces filopodia, and modulates migration of infected lymphocytes. Journal of virology. 2010;84:2282–93. 10.1128/JVI.02230-09 . 20015995 PMC2820911

[89] Park I-W, He JJ. HIV-1 Nef-mediated inhibition of T cell migration and its molecular determinants. Journal of leukocyte biology. 2009;86:1171–8. 10.1189/jlb.0409261 . 19641037

[90] Kotler DP, Gaetz HP, Lange M, Klein EB, Holt PR. Enteropathy associated with the acquired immunodeficiency syndrome. Annals of internal medicine. 1984;101:421–8. 10.7326/0003-4819-101-4-421 . 6476631

[91] Kotler DP, Shimada T, Snow G, Winson G, Chen W, Zhao M, et al Effect of combination antiretroviral therapy upon rectal mucosal HIV RNA burden and mononuclear cell apoptosis. AIDS (London, England). 1998;12:597–604. 10.1097/00002030-199806000-00008 . 9583599

[92] Vitkovic L, Konsman JP, Bockaert J, Dantzer R, Homburger V, Jacque C. Cytokine signals propagate through the brain. Molecular psychiatry. 2000;5:604–15. 10.1038/sj.mp.4000813 . 11126391

[93] Blamire AM, Anthony DC, Rajagopalan B, Sibson NR, Perry VH, Styles P. Interleukin-1beta -induced changes in blood-brain barrier permeability, apparent diffusion coefficient, and cerebral blood volume in the rat brain: a magnetic resonance study. The Journal of neuroscience: the official journal of the Society for Neuroscience. 2000;20:8153–9. 10.1523/JNEUROSCI.20-21-08153.2000 . 11050138 PMC6772751

[94] Oliveira SHP, Canetti C, Ribeiro RA, Cunha FQ. Neutrophil migration induced by IL-1beta depends upon LTB4 released by macrophages and upon TNF-alpha and IL-1beta released by mast cells. Inflammation. 2008;31:36–46. 10.1007/s10753-007-9047-x . 17874178

[95] Russell RA, Chojnacki J, Jones DM, Johnson E, Do T, Eggeling C, et al Astrocytes Resist HIV-1 Fusion but Engulf Infected Macrophage Material. Cell Rep. 2017;18(6):1473–83. Epub 2017/02/09. 10.1016/j.celrep.2017.01.027 28178524 PMC5316642

[96] Guan-Han L, Lisa H, Avindra N. Astrocytes as an HIV Reservoir: Mechanism of HIV Infection. Current HIV Research. 2016;14(5):373–81. 10.2174/1570162x14666161006121455 27719663 PMC11345863

[97] Hoxie JA, LaBranche CC, Endres MJ, Turner JD, Berson JF, Doms RW, et al CD4-independent utilization of the CXCR4 chemokine receptor by HIV-1 and HIV-2. Journal of reproductive immunology. 1998;41(1–2):197–211. Epub 1999/04/23. 10.1016/s0165-0378(98)00059-x . 10213311

[98] Liu Y, Liu H, Kim BO, Gattone VH, Li J, Nath A, et al CD4-Independent Infection of Astrocytes by Human Immunodeficiency Virus Type 1: Requirement for the Human Mannose Receptor. J Virol. 2004;78(8):4120–33. 10.1128/JVI.78.8.4120-4133.2004 15047828 PMC374297

[99] Gray LR, Roche M, Flynn JK, Wesselingh SL, Gorry PR, Churchill MJ. Is the central nervous system a reservoir of HIV-1? Current opinion in HIV and AIDS. 2014;9(6):552–8. Epub 2014/09/10. 10.1097/COH.0000000000000108 25203642 PMC4215931

[100] Eugenin EA, Berman JW. Gap Junctions Mediate Human Immunodeficiency Virus-Bystander Killing in Astrocytes. J Neurosci. 2007;27(47):12844–50. 10.1523/JNEUROSCI.4154-07.2007 18032656 PMC2117380

[101] Ton H, Xiong H. Astrocyte Dysfunctions and HIV-1 Neurotoxicity. Journal of AIDS & clinical research. 2013;4(11):255 Epub 2014/03/04. 10.4172/2155-6113.1000255 24587966 PMC3938291

[102] Mediouni S, Marcondes MC, Miller C, McLaughlin JP, Valente ST. The cross-talk of HIV-1 Tat and methamphetamine in HIV-associated neurocognitive disorders. Frontiers in microbiology. 2015;6:1164 Epub 2015/11/12. 10.3389/fmicb.2015.01164 26557111 PMC4615951

[103] Liu X, Kumar A. Differential signaling mechanism for HIV-1 Nef-mediated production of IL-6 and IL-8 in human astrocytes. Scientific reports. 2015;5:9867 Epub 2015/06/16. 10.1038/srep09867 26075907 PMC4467202

